# A challenging complication following SARS-CoV-2 infection: a case of pulmonary mucormycosis

**DOI:** 10.1007/s15010-020-01561-x

**Published:** 2020-12-17

**Authors:** Daniela Pasero, Silvana Sanna, Corrado Liperi, Davide Piredda, Gian Pietro Branca, Lorenzo Casadio, Raffaella Simeo, Alice Buselli, Davide Rizzo, Francesco Bussu, Salvatore Rubino, Pierpaolo Terragni

**Affiliations:** 1 Intensive Care Unit, Emergency Department, AOU Sassari, Sassari, Italy; 2 Department of Surgical Medical and Experimental Medicine, University of Sassari, Viale San Pietro 43, 07100 Sassari, Italy; 3 Microbiology and Virology Units, AOU Sassari, Sassari, Italy; 4 Otolaryngology Division, AOU Sassari, Sassari, Italy; 5 Department of Biomedical Sciences, University of Sassari, Sassari, Italy

**Keywords:** SARS-Cov-2, Mucormycosis, Opportunistic infections

## Abstract

Severe acute respiratory syndrome coronavirus 2 infection might induce a significant and sustained lymphopenia, increasing the risk of developing opportunistic infections. Mucormycosis is a rare but severe invasive fungal infection, mainly described in immunocompromised patients. The first case of a patient diagnosed with coronavirus disease (COVID-19) who developed a pulmonary mucormycosis with extensive cavitary lesions is here reported. This case highlights how this new coronavirus might impair the immune response, exposing patients to higher risk of developing opportunistic infections and leading to worse outcomes.

## Introduction

Mucormycosis is a rare but severe invasive fungal infection occurring mainly in immunocompromised patients, especially in individuals diagnosed with uncontrolled diabetes mellitus or haematological malignancies, as well as in previously healthy subjects with open wounds contaminated by Mucorales [1–3]. Patients with severe acute respiratory syndrome coronavirus 2 (SARS-CoV-2) infection might develop coronavirus disease (COVID-19), which can be associated to significant and sustained lymphopenia compromising the immune system, especially in the most severe cases [4–6]. Some authors described that a significant decrease in lymphocyte count and an increase of neutrophil count together with an inflammatory storm, occur more frequently in patients who developed severe COVID-19 and co-infections [4]. This report describes the first case of a patient with SARS-CoV-2 infection who developed a cavitary pulmonary mucormycosis.

## Case report

**Fig. 1:**
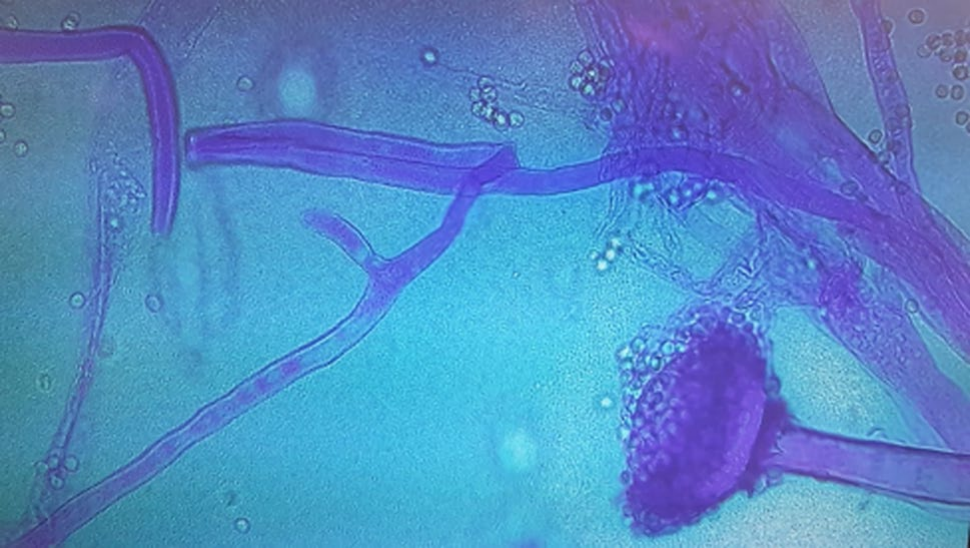
Microbiological sample from bronchial aspirate: morphology of *Rizhopus *spp.. Lactophenol cotton blue preparation showed aseptate broad hyphae, sporangia and sporangiospores

**Fig. 2:**
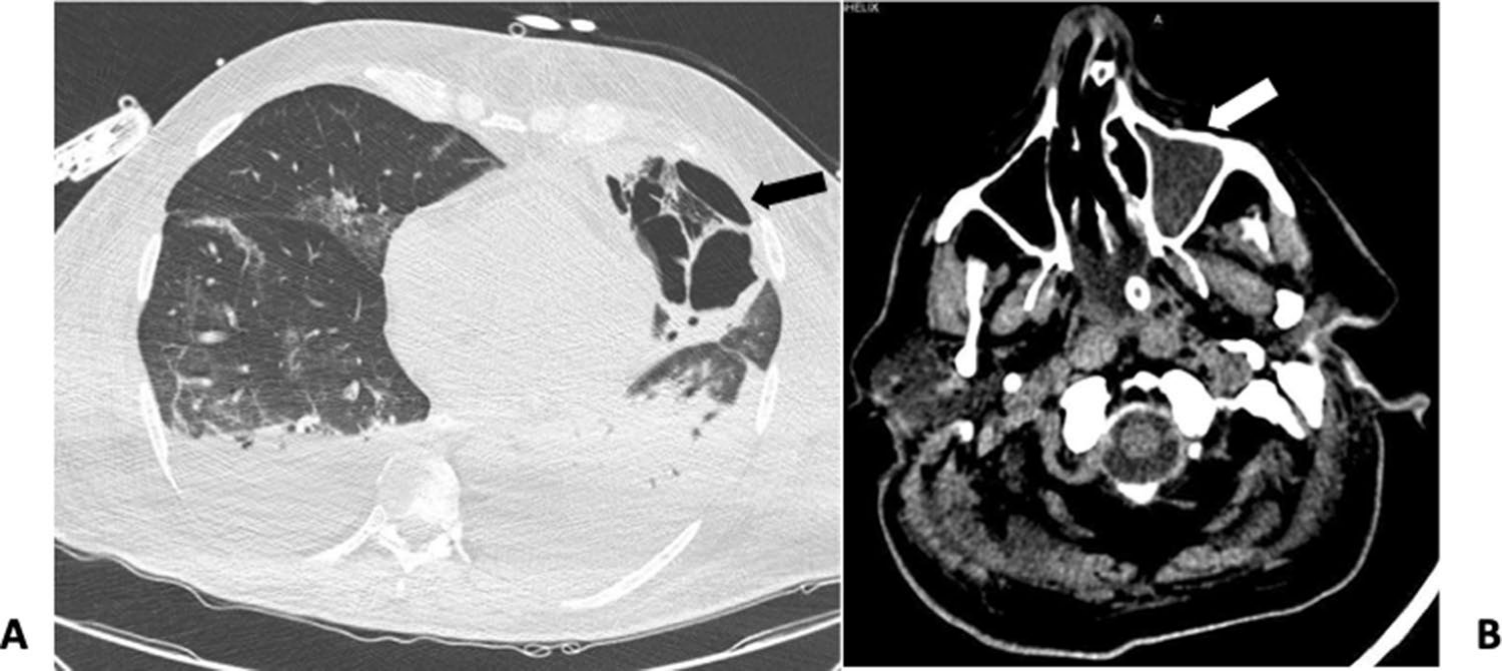
**a** Thoracic computed tomography (CT) scan showed buried cavitary lesions in the left lung; **b** cranial CT scan showed corpuscular material in the left maxillary sinus

A 66-year-old male patient was admitted to ICU at the University Hospital, in Sassari, Italy, on March 26, 2020, with a diagnosis of SARS-CoV2 infection. Due to a rapid and progressive deterioration of oxygenation, the patient was intubated after a short period of non-invasive respiratory support. He had a history of arterial hypertension treated with ACE-inhibitors and had recently been diagnosed with urinary tract infection. The beginning of COVID-19 symptoms reportedly started one week before admission. A therapy with hydroxychloroquine and lopinavir-ritonavir was administered for the first 10 days. At ICU admission, the patient was deeply sedated, underwent protective mechanical ventilation, according to the new evidence described for such pulmonary damage phenotype, to avoid ventilator-induced lung injury [7, 8] (tidal volume = 6–7 ml kg^−1^ *PBW, positive end expiratory pressure (PEEP) = 12 cmH_2_O; PaO_2_/FiO_2_ = 262); he also required circulatory support with vasopressor (norepinephrine = 0.2 mcg kg^−1^ min). In addition, the patient had multiple organ dysfunction syndrome with sequential organ failure assessment (SOFA) [9] = 14. A few days after admission, respiratory parameters progressively worsened: a lower oxygenation (PaO_2_/FiO_2_ = 174), increase of radiological infiltrates and a parenchymal thickening of the left lower lobe were observed. Table 1 describes the main clinical and laboratory data, which include crucial biomarkers reported at ICU admission and during the whole hospitalization. The patient did not present with fever, the white blood count (WBC) and neutrophils increased while lymphocytes progressively decreased (lymphocytes [Nadir] = 400 μl^−1^; neutrophil/lymphocyte [N/L] ratio = 19.2). As C-reactive protein CRP (= 14.3 mg dl^−1^) and procalcitonin PCT (= 6.91 ng ml^−1^) values were elevated he was administered empirical antibiotic therapy (piperacillin-tazobactam 18 gr IV infusion, 24 h a day and levofloxacin 700 mg a day). As a result of a reduced kidney function, a renal replacement therapy (RRT) was required for several days. However, after 2 weeks of empirical therapy, neither were pathogens isolated on microbiological work-up of samples, nor an improvement of oxygenation (PaO_2_/FiO_2_ = 130) was observed. Inflammatory biomarkers showed higher values (CRP = 25 mg dl^−1^; PCT = 13.1 ng ml^−1^) and a further worsening of pulmonary infiltrates with an increase of parenchymal thickening of the whole left lung was observed by imaging techniques. Empirical therapy was replaced by meropenem 1 gr IV infusion every 24 h (dose-adjusted for renal dysfunction) and linezolid 600 mg every 12 h and a bronchial aspirate (BAS) was repeated to confirm SARS-CoV-2 and detect any additional co-infections. Two weeks after ICU admission a surgical tracheostomy was performed at bedside for prolonged mechanical ventilation. [10] A first BAS negative for SARS-CoV-2 was obtained, while the second BAS tested for co-infections showed rapidly growing cotton-candy like colonies on sabouraud dextrose agar (SDA) at 30 °C. Microscopic examination with lactophenol cotton blue preparation showed aseptate broad hyphae, sporangia containing sporangiospores. The mould was identified as *Rhizopus *spp. (Fig. 1) based on the phenotype features, however no susceptibility test could be performed at our laboratory. A cranial and thoracic computed tomography (CT) scan was performed to search for specific lesions. Non-encephalic lesions were found, opacification of the left maxillary sinus and thickening with sclerosis of sinus walls were observed and the thoracic scans were suggestive of buried cavitary lesions in the lingula of the left lung upper lobe (Fig. 2). Treatment with liposomal Amphotericin B, 5 mg kg^−1^ IV was commenced, according to guidelines and after discussing with an infectious disease consultant [11, 12]. A second and a third BAS were consecutively positive for *Rhizopus *spp*.* A biopsy of the left maxillary sinus was performed to find the source of mould infection, but samples were positive only for *Candida glabrata*. A transbronchial biopsy was excluded because of severe hypoxia and high risk of airway bleeding. A probable pulmonary mucormycosis was then hypothesized. After 16 days of antifungal treatment, a slow improvement of gas exchange was noticed. Since the bronchoalveolar lavage (BAL) was still positive for *Rhizopus *spp., a surgical evaluation was requested, and thoracic CT scan was repeated. The scan revealed a rupture of the cavities previously observed in the pleural space and bilateral pleural effusion was observed. Therefore, a thoracentesis was performed at bedside. Samples of pleural effusion were tested for microbiological and histopathological examination, but neither moulds nor other fungi were isolated. At that stage, surgery was not performed, being regarded as high-risk intervention. Finally, 40 days after ICU admission and 20 days after the beginning of liposomal Amphotericin B treatment, even though *Rhizopus spp* growth was still observed in BAL samples, the patient clinically improved and recovered from lymphopenia (lymphocyte = 1,800 μl^−1^, *N*/*L* = 3.5). Since persistency of the positive culture on BAL for *Rhizopus spp* was still occurring, a surgical consultation was planned to eradicate the necrotic lesions from the left lung. At the same time, the antifungal treatment was shifted to Isavuconazole and the liposomal Amphotericin B treatment was suspended [13]. However, since following thoracentesis the oxygenation considerably improved (PaO_2_/FiO_2_ > 300), surgery was postponed and also the surgeon evaluated the patient as being at extremely high risk for surgery. Sedation was then suspended, and the patient began a ventilatory weaning process. Furthermore, an improvement of other organ functions was observed, such as in the kidneys, as the urine volume started to increase gradually. The following week, a new clinical deterioration was observed, showing fever, an increase of PCT, severe haemodynamic instability and worsening of kidney and liver functions, probably due to a bacterial co-infection. Although an antifungal treatment with Isavuconazole was maintained together with a prompt empirical antibiotic therapy, the patient died at day 62 after ICU admission due to refractory shock and liver failure. Unfortunately, the autopsy could not be performed due to the lack of a negative pressure room required to execute safe procedures.

**Table 1:** Daily clinical and laboratory variables during ICU stay

Variables	Reference range	Day 1 admission			Day 3			Day 7		Day 10					Day14		Day21			Day 28			Day 40
SOFA		13			16			16		16					17		16			15			13
White blood counts (per μl)	(4800–10,800)	5,700			11,710			9990		8760					17,070		27,930			23,750			10,710
Lymphocytes(per μl)	(900–5200)	1000			700			800		400					800		1,200			800			1800
Neutrophils (per μl)	(1900–8000)	4,300			10,300			8,500		7700					12,600		23,600			21,100			6300
N/L		4.3			14.7			10.6		19.2					15.7		19.6			26.3			3.5
CRP (mg/dl)	(0–1)	17.7			31.9			18.6		14.3					25		17.1			8.65			8.80
Procalcitonin(ng/ml)	(0–0.5)	1.9			13			7.92		6.91					13.1		10			6.06			5.75
Glycemia (mg/dl)	(60–99)	124			96			96		70					92		96			86			96
D-dimer (mg/l)	(0–0.5)	1.9			4.3			0.8		1					2.6		2.7			6.1			7.64
Ferritin (ng/ml)	(26–388)	3216			3755			2012		1395					2128		2737			2084			–
NT-proBNP (pg/ml)	(0–125)	653			2147			2869		2801					3028		12,541			26,116			19,692
PaO_2_/FiO_2_		190			197			208		174					130		161			189			260
SARS-CoV-2 RT-PCR					Positive			Positive		Positive					Positive		Negative Negative after 24 h			Negative			
BAS/BAL															*Rhizopus* spp.		*Rhizopus* spp.			R*hizopus* spp.			*Rhizopus* spp.
Pleural effusion	Histo-pathology																						Negative
	Microbio-logy																						Negative

*SOFA* sequential organ failure assessment, *N/L* neutrophils/lymphocyte ratio, *CRP* C-reactive protein

## Discussion

SARS-CoV-2 infection might alter the immune system by affecting T lymphocytes, particularly CD4+ and CD8+ T cells, which might be highly involved in the pathological process of COVID-19 infection [4]. The significant reduction of the absolute number of lymphocytes and specifically of T cells described in the most severe COVID-19 cases, is associated with the worst outcome and might expose patients to a higher risk of developing opportunistic infections [3, 4]. Mucormycosis is a fungal infection caused by a group of opportunistic moulds, i.e., mucormycetes [2, 3]. This infection is generally caused by an impairment of bronchial alveolar macrophages, but a role of T-cells was described as part of the adaptive immune system. Potenza et al., in a brief report on a group of haematological patients who suffered from mucormycosis described *Mucorales*-specific T-cells (CD4+ and CD8+), that were active against *Mucorales* by producing cytokines, such as IL-4, IL-10, IL-17 and IFN-γ, which could directly damage *Mucorales hyphae* [14]. The authors observed *Mucorales*-specific T-cells only in patients affected by invasive mucormycosis and they concluded that they could be a useful surrogate diagnostic marker of an invasive fungal disease and they might contribute to control the invasive fungal infection by *Mucorales*. We might speculate that lymphopenia could increase the risk of developing an invasive mucormycosis, while the recovery of lymphocytes count could improve the adaptive immune system and induce the production of *Mucorales*-specific T-cells, which might have a role in controlling the invasive infection. Unfortunately no lymphocyte subset typing could be performed by our haematological laboratory due to the fact thatat the beginning of the outbreak safe procedures to manage samples from patients affected by COVID-19 had not been implemented yet.

Different organs might be involved, the most frequently affected of which are the lungs, being the second most common manifestation (58%) with a mortality rate up to 80% due to its aggressive clinical course [2].

The high mortality rate described in pulmonary localization might be related to delays in diagnosis, to an unbalanced immune system and a poor host response, as well as to the complexity of the treatment that includes a combination of antifungal therapy and a high-risk surgical intervention [3, 4, 15, 16]. In the present case, a surgical intervention was not performed because a clinical improvement was observed in lung function, and surgeons considered the patient at extremely high risk for surgery.

Generally, mucormycosis affects immunocompromised patients: as a matter of fact, a recent systematic review by Jeong et al. showed that solid organ transplantations and neutropenia, commonly reported in patients affected by haematological malignancies, were the only independent risk factors for pulmonary mucormycosis [17]. Furthermore, SARS-CoV-2 infection itself might trigger an alteration of the immune system [4] and this is the first reported case of opportunistic co-infection caused by *Rhizopus spp* involving lungs with an extensive parenchymal damage.

Recently in a retrospective study, Koehler et al. analysed a cohort of patients admitted to ICU due to COVID-19 showing moderate to severe acute respiratory distress syndrome (ARDS) who developed invasive pulmonary aspergillosis as a consequence of the immune-paralysis related to SARS-CoV-2 infection [18]. Similarly, in the present case neither corticosteroids nor immunosuppressant therapies were administered, but the patient showed a severe form of COVID-19 with multiple organ dysfunctions and a significant and sustained lymphopenia with N/L ratio alteration; the latter has been recently described to be highly associated with the most severe clinical presentation and the worst outcome [4]. Therefore, it might be suggested that SARS-CoV-2 infection by itself can induce an immunosuppressive state that exposes the patient to the risk of developing opportunistic infections, such as moulds. These kind of infections by themselves are associated with the worst outcome, especially when the immune system response does not improve. However, when the immune system recovers, opportunistic infections might be controlled [16], as the present case shows, when an improvement of its clinical symptoms, specifically of the respiratory dysfunction, was observed. As a matter of fact, the patient’s oxygenation began to improve when lymphocytes increased and N/L ratio decreased, and the pulmonary cavitary lesion opened into the pleural space. Although moulds could not be isolated in the pleural effusion, surgical exploration to remove necrotic lesions should be considered to eradicate the mould infection and improve the patient’s outcome [15].

The European Confederation of Medical Mycology Mucormycosis Guidelines strongly suggest an early surgical treatment to remove the infected tissue (either through local debridement or complete resection) in addition to systemic antifungal treatment [11].

Which is the lesson learnt from the present case? When no improvement of clinical symptoms is shown following a wide spectrum empirical treatment, and when an impaired immune response induced by SARS-CoV-2 is observed, opportunistic infections, such as the mould infection shown in the present case, should be investigated as a potential causative agent. As a result, the antimicrobial therapy should include antifungal agents and, when data suggest a mucormycosis, a pharmacological treatment should be associated whenever possible with a surgical intervention to eradicate mould-associated necrotic lesions.

In conclusion, SARS-CoV-2 infection is regarded as a cause of severe immunosuppression that might compromise the host response and increase the risk to develop opportunistic infections, including those caused by moulds, leading to higher risk of negative outcomes in the case of delayed diagnosis and inadequate treatment.
